# Introductory Lectures

**Published:** 1845-01

**Authors:** 


### INTRODUCTORY LECTURES.

A number of Introductory Lectures, delivered at the opening of the various medical schools of the country, during the last autumn, have been laid on our table. The first we received was from Professor Harrison, of Cincinnati. His subject is "*The Formation of Professional Character,*" and is discussed with the earnestness of a man conscientiously devoted to the interests of his profession and of society. If we were to object to the lecture in any way, it would be to the style, which is, perhaps, somewhat verbose; but the matter is good, and the spirit excellent. We give a specimen:

"Industry is the primal fountain of all prosperity. The savage is indolent, and he is poor. Civilized man owes his comforts and luxuries;—his might and glory, to the ceaseless toil of his intellect and hands. Educing from the hidden resources of nature vast elements of power, the genius of man triumphs over the wants and weakness of his condition, and renders the very powers which threatened his existence contributory to his well-being.

"Destitute of industry—of a well-sustained, systematic exertion of our mental powers, nothing permanently useful can be attained in our profession. Accident, or rash experiment, may hit on some valuable discovery but of what avail is such a disclosure on the part of nature, if the patient labor of man does not make those applications of the discovery which render it available? The truth or fact discovered by accident or rash experiment, must be lifted up out of the isolation in which it stands and be made to blend with other truths or facts, and thus become instinct of new related tendencies. Patient toil of mind alone can discern these tendencies, and by giving a wider range of direction to the original discovery indefinitely expand the sphere of its agency.

"From the hour when the student takes a medical work in hand, through all the stages of his professional studies and researches, up to his brightest achievements of remedial skill, and along the whole progress of his career, an unremitting irrepressible exertion of his powers is required.

"Patience, as an habitual state of mind, must, with presiding authority, ever accompany the physician. When a student, there is a constant need of patience;—when young, in the active duties of the profession, patience is required—and in the busy employments of a large practice, patience must be exercised. Patience of preparation, the student should possess; patience of probation the young physician must have; and patience of temper, amidst the privations of repose and social enjoyments, and in the exhaustion of his mental and bodily strength, must be the ever-present support of the older practitioner."

Professor Draper, of the University of New York, has produced a lecture, "*On the Relations of Atmospheric Air to Animals and Plants*," of very great beauty. In parts it is highly elaborate, and it abounds in poetry, as well as in science, as the following paragraph testifies:

"From this point of view, therefore, I look upon the vegetable world as an embodiment of the action of ethereal agents. A tree, when covered with blossoms in the spring, or laden with fruit in the autumn, is a resultant of the play of those active forces which have been emitted by the sun; an expression of what has been done by vibratory movements operating on ponderable molecules. As soon as the young plant has exposed itself to the solar beam, growth rapidly begins to take place, and organized matter to be condensed from the air, and now a green color is developed, and the stem elongates and leaves are put forth. In carrying forward all those multiplied operations which have ended in these events, its leaves and its stem have gone upward in search of light—light which has symmetrically arranged their parts and furnished their substance. But these general views are far from giving us an accurate idea of the forces that have been expended or the motions which have been executed in producing the result we contemplate. A forest tree, from its magnitude, rising perhaps a hundred feet from the ground, and spreading its branches over hundreds of square yards, may impress us with a sense of sublimity; a section of its stem might assure us that it had lived for a thousand years, and its total weight could only be expressed by tons. An object like this may indeed call forth our admiration, but that admiration is expanded into astonishment, when we come to consider minutely the circumstances which have been involved in producing the result. If we conceive a single second of time, the beat of a pendulum, divided into a million of equal parts, and each one of those inconceivably brief periods divided again into a million of other equal parts—a wave of yellow light during one of these last small intervals has vibrated five hundred and thirty-five times. And now that yellow light is the agent which has been mainly involved in building up the parts of the tree, in fabricating its various structures, and during every one of a thousand summers, from sunrise to sunset the busy rays have been carrying on their operation,—who then can conceive when in the billionth of a second such enormous numbers of movements are accomplished, how many have been spent in erecting an aged forest oak? Who also can conceive the total amount of force employed from century to century in arranging the vegetation of the surface of the globe?"

Professor Childs delivered a lecture before the medical class of Willoughby University which is marked by vigorous and just thought, and a pure, simple, perspicuous style. His subject is "*General Principles in Medicine.*" We make room for a single extract:

"This leads me to notice *two classes* of educated physicians, (and of the educated only I speak)—and the striking contrast they exhibit. In the one class, the physician adopts an *exclusive system* and practices by *rule.* He is confined to a specific direction, which, in the nature of things, must be limited in its application. All must be stretched or shortened to the same Procrustean bed—all must take the same remedy—investigation is proscribed, and discrimination is heresy. If timid, his practice must be inefficient; if bold, he strikes with herculean club, but uncertain aim, a hazardous and probably fatal blow. He must grope in darkness. The other arranges the *facts* of medicine, and deducing therefrom by a legitimate induction, *general principles,* makes these the sole guides in his practice. In these, too, he finds a *true standard,* the great desideratum in medicine, and to this test brings all theory, and all modes of practice—a righteous decision awaits the trial. In the Cullenian, Brunonian and Broussaian systems—systems which have exerted a mighty sway over the profession at different periods in the history of science, which have divided the medical world into sects, and perpetuated the names of their authors, he finds the most opposite and contradictory doctrines. They cannot all be true. Arraign them at the bar of general principles, and what is the verdict rendered? In all, *some truth* and *much errer* are the ingredients of the composition. The chaff must be winnowed from the wheat. What of truth in each will be retained, and the proof of the true is demonstrated from its consistency with itself and its harmony with all known truth. *His principles* will include every remedial agent ever found useful in the treatment of disease. From the harmless leeches and gum water of Broussais, to the ready lancet and the energetic calomel of Rush—from the depletions of Sydenham to the stimulants of Brown—from the cold water of Hydropathy, to the hot water of Sangrado—none will be proscribed from *his* system. And, think you that the scientific and discriminating physician I have described—the man of principle—will not measure swords with his exclusive competitor? Nay, he will vanquish him *with his own weapons!*

The lecture of Austin Flint, M.D., Professor of the Institutes of Medicine, in the Rush Medical College, at Chicago, will compare with the best of this class of productions. The author is a scholar, and a polished and elegant writer. We regret that we have no room for an extract. Y.

